# Impact of COVID-19 Pandemic on the Overall Diagnostic and Therapeutic Process for Patients of Emergency Department and Those with Acute Cerebrovascular Disease

**DOI:** 10.3390/jcm9123842

**Published:** 2020-11-26

**Authors:** Hansol Chang, Jae Yong Yu, Sun Young Yoon, Sung Yeon Hwang, Hee Yoon, Won Chul Cha, Min Seob Sim, Ik Joon Jo, Taerim Kim

**Affiliations:** 1 Department of Emergency Medicine, Samsung Medical Center, Sungkyunkwan University School of Medicine, 115 Irwon-ro Gangnam-gu, Seoul 06355, Korea; briquet90@naver.com (H.C.); wildhee@gmail.com (H.Y.); docchaster@gmail.com (W.C.C.); minsub01.sim@samsung.com (M.S.S.); Ikjoonjo@smasung.com (I.J.J.); 2 Department of Digital Health, Samsung Advanced Institute for Health Science & Technology (SAIHST), Sungkyunkwan University, 115 Irwon-ro Gangnam-gu, Seoul 06355, Korea; icalust@gmail.com (J.Y.Y.); emtyoon92@gmail.com (S.Y.Y.); gerup@hanmail.net (S.Y.H.); 3 Health Information and Strategy Center, Samsung Medical Center, 81 Irwon-ro Gangnam-gu, Seoul 06351, Korea

**Keywords:** length of stay, emergency medical service, COVID-19, pandemic, process

## Abstract

(1) Background: During a pandemic, patients and processes in the emergency department (ED) change. These circumstances affect the length of stay (LOS) or degree of crowding in the ED. The processes for patients with acute critical illness, such as cerebrovascular disease (CVD), can be also delayed. Using the process mining (PM) method, this study aimed to evaluate LOS, ED processes for CVD, and delayed processes during the coronavirus disease 2019 (COVID-19) pandemic. (2) Methods: Data were collected from the Clinical Data Warehouse of a medical center. Phase 1 included patients who visited the ED before the COVID-19 outbreak. In Phase 2, post-COVID-19 ED patients were divided into the COVID-19 tested group (CTG) and COVID-19 not tested group (CNTG) according to whether polymerase chain reaction test was performed. We analyzed patients’ ED processes before and after COVID-19 using the PM method. We analyzed patients with acute CVD separately to determine whether the process and LOS of patients with acute critical illness were changed or delayed. (3) Results: After the COVID-19 outbreak, the overall LOS was delayed and all processes in CTG patients were delayed. Registration to triage and triage were delayed in both CTG and CNTG patients. The brain imaging process for CTG patients with acute CVD was also delayed. (4) Conclusion: After a pandemic, some processes were changed, new processes were developed, and processes for patients with acute CVD who needed proper time management were not exempted.

## 1. Introduction

Several pandemics have occurred in the past, such as the severe acute respiratory syndrome, Middle East respiratory syndrome, and influenza [1,2]. Currently, the coronavirus disease 2019 (COVID-19) has emerged as a pandemic. During a pandemic, the hospital system should be changed, owing to an increase in the demand for medical resources, and the emergency department (ED) is not an exception [3,4]. In this situation, patients and the processes of ED change, and these circumstances affect the length of stay (LOS) or degree of crowding in the ED [4].

LOS in the ED is closely related to the outcome of patients in the ED [5,6,7]. Even in patients who need acute care, crowding can delay the total LOS [8]. Therefore, if a pandemic increases the LOS of ED patients who need acute care, it will result in poor outcomes in patients with acuity. Some studies have also shown that the incidence of in-hospital cardiac arrest (IHCA) increased as the LOS in the ED increased [9].

If a pandemic affects an acutely severe patient’s process and LOS, which is, in some cases, time critical, then it can also change the outcome of those patients by worsening the delay of proper evaluation and management. For example, acute cerebrovascular disease (CVD), time to make diagnosis, and treatment are critical for patient outcome [10,11]. For acute CVD, especially for stroke syndrome, fast and proper brain imaging has been reported [10,12,13]. The American Heart Association (AHA) guidelines recommend 6–24 h of undergoing computed tomography (CT) or magnetic resonance imaging (MRI) [14]. Delays in these processes can be critical for the diagnosis of acute CVD patients [15]. Therefore, processes for patients with acute critical illness, such as CVD, require special attention to save diagnosis and treatment time even during a pandemic.

To evaluate the LOS and the delayed process, the process mining (PM) method was applied in this study. Using PM, it is possible to compare process changes by environment or situation changes, for example, in this study, process changes during a pandemic situation [16,17,18,19,20]. Some studies have used PM to assess hospital outpatient clinic processes; however, a comparative analysis of LOS in the ED before and after COVID-19 using PM has not been well established [17,19].

This study investigated the general ED patient process and LOS in the ED before and after the COVID-19 pandemic by timeline. In addition, this study also compared the process and outcome in patients with acute stroke or hemorrhage without trauma according to whether the COVID-19 polymerase chain reaction (PCR) test was performed. By doing so, the study will assist in setting further strategies to manage patients, especially for those who need acute management in the pandemic era.

## 2. Methods

### 2.1. Data Collection

This retrospective study was conducted in the ED of a tertiary hospital located in a metropolitan city. The hospital has approximately 1960 inpatient beds. The annual number of patients in the ED is approximately 80,000. This study included patients who visited the ED from 1 February 2019 to 31 July 2019 and 1 February 2020 to 31 July 2020.

**Figure 1:**
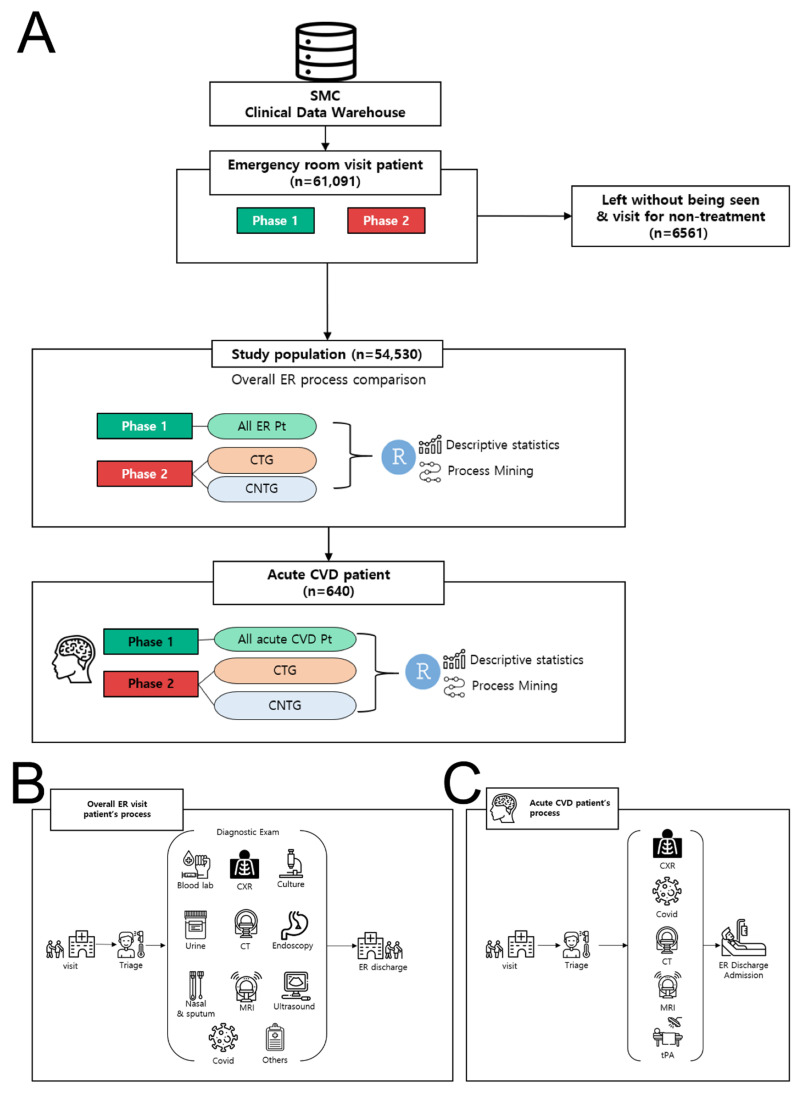
Flowchart of the study. (**A**) Phase 1 included patients who visited the Emergency Department (ED) from 1 February 2019 to 31 July 2019. Phase 2 included patients who visited the ED from 1 February 2020 to 31 July 2020. Phase 2 was divided into the “coronavirus disease 2019 (COVID-19) Tested Group” (CTG) and “COVID-19 Not Tested Group” (CNTG). CTG included patients who underwent the COVID-19 PCR test, and CNTG included patients who did not undergo the COVID-19 PCR test. (**B)** Process analysis of all overall ED patients. (**C**) Process analysis of patients with acute cerebrovascular disease. COVID-19: coronavirus disease 2019; ED: emergency department, SMC: Samsung Medical Center, ER: Emergency Room, CTG: COVID-19 tested group, CNTG: COVID-19 not tested group.

We divided the study periods into two phases. Phase 1 included patients who visited the ED from 1 February 2019 to 31 July 2019. Phase 2 included patients who visited the ED from 1 February 2020 to 31 July 2020. Phase 2 was divided into “COVID-19 Tested Group” (CTG) and “COVID-19 Not Tested Group” (CNTG). CTG included patients who underwent the COVID-19 PCR test, and CNTG included patients who did not undergo the COVID-19 PCR test. We excluded patients without information on time in registration, execution, and reading of laboratory tests. We further analyzed CVD patients in the same manner as above and divided them into Phase 1, CTG and CNTG. The process of selecting patients is shown in Figure 1.

The institutional review board of the hospital approved this study. Informed consent was waived because this was a retrospective, observational, and de-identified study (SMC 2020-08-184).

### 2.2. Indication of COVID-19 PCR Test

The COVID-19 PCR test was performed in accordance with the center’s policy for patients with at least one of the following symptoms: fever, cough, sputum production, rhinorrhea, diarrhea, arthralgia, and dyspnea. Patients who stayed for more than 24 h at another hospital or who stayed in a crowded public housing facility for more than 24 h also underwent the COVID-19 PCR test. Finally, patients with a contact history to individuals with confirmed COVID-19 underwent the COVID-19 PCR test. The center collected this information through a questionnaire prior to registration and double-checked such information in triage to sort these patients (Supplementary Figure S1). In addition, patients who had abnormal chest radiography or chest CT findings underwent the COVID-19 PCR test, even after they were classified as low-risk patients and were not indicated for COVID-19 PCR test in triage.

### 2.3. Selection of Presumed Acute Cerebrovascular Patients

We analyzed patients with acute CVD separately to determine whether the process and LOS of severely acute patients were changed or delayed. We separated these patients using the International Statistical Classification of Diseases and Related Health Problems, 10th revision (ICD-10) codes that are specific for acute CVD (I210, I211, I212, I213, I214, I219, I6300, I6301, I6302, I6308, I6309, I6310, I6311, I6312, I6318, I6319, I6320, I6321, I6322, I6328, I6329, I6330, I6331, I6332, I6333, I6338, I6339, I6340, I6341, I6342, I6343, I6348, I6349, I6350, I6351, I6352, I6353, I6358, I6359, I636, I638, I639, I64, I610, I611, I612, I613, I614, I615, I616, I618, I619, I620, I621, I629, I600, I601, I602, I603, I604, I605, I606, I607, I608, I609). We also performed the same analysis with the total number of patients in the ED, such as using PM, in the case of acute cerebrovascular patients, and analyzed the length of time of each process. In the case of acute cerebrovascular patients, we focused on brain imaging processes such as brain CT and MRI.

### 2.4. Data Extraction and Preparation

In hospitals using electronic medical record (EMR), log data such as patient demographics and treatment information are available; event log refers to a set of events that are recorded in the context of a process. Patient clinical information, ED visit information, examination prescription information, and time of other evaluation tests were extracted from the Clinical Data Warehouse Darwin-C of Samsung Medical Center for this study. Patient information included age, sex, systolic blood pressure, diastolic blood pressure, pulse rate, respiratory rate, body temperature at arrival, oxygen saturation, Korean triage and acuity scale, mental status, and mode of arrival. In addition, as outcome information, we collected discharge information, including discharge to home, ED death, transfer, and admission. We also recorded the place where admission information was collected, which included the intensive care unit (ICU) and general ward (GW). The major time information used for PM included ED visit time, initial nurse assessment time, registration, laboratory test time, imaging test time, CT, MRI, electrocardiography (ECG) test time, COVD-19 PCR test time, radiologic intervention time, and discharge time.

### 2.5. Process Mining

We describe the methodology in three parts for analyzing the change in the ED process before and after the COVID-19 pandemic using PM. PM is a methodology that focuses on extracting knowledge from database event logs. The major purpose of PM is to discover and monitor the conformance of the process. There are three essential key pieces of information for the execution in the process analysis: patient ID, timestamp, activity. An event log can be viewed as a set of traces that contains the sequence of each activity. We could identify the main process, bottleneck of the process, and total time spent between the activities.

Figure 1 shows the framework for the overall process analysis. It consists of data extraction and preparation, creation of a process model, and interpretation. Using PM, we calculated the total spent time from visit to discharge and from one process to another [18,21]. We also analyzed patient distribution from one process to another [18,21]. Based on these results, we drew a figure of a process model figure that showed the total process flow during ED stay in each group [22,23]. Expressing every test into the process model activity can generate the spaghetti model, which is complex and difficult to figure out. Therefore, to reduce the complexity, we categorized the tests. We divided laboratory tests by sample: blood, nasal, and sputum. Blood culture was categorized separately. Imaging test was divided by equipment: CT, MRI, X-ray, and ECG. The COVID-19 PCR test was expressed separately.

Several tools are available for PM such as “proM” and “Disco,” and we used the “bupaR” framework in R Studio to discover the process model of ED visit in the event log.

### 2.6. Interpretation

Finally, it was essential to have in-depth discussion with medical experts in the ED. We compared the route and the time derived from the PM results.

### 2.7. Statistical Analysis

We used two main analyses for comparison. First, we compared the time and the number of patients in Phase 1 (February–June 2019), and Phase 2 was divided as CTG and CNTG (February–June 2020) among patients with severe disease. Second, subgroup analysis for acute CVD patients was conducted. To clarify the results of the process, we used PM visualization and a chord diagram. For statistical analysis, we used R, version 3.6.0. Continuous variables are presented as medians and interquartile range (IQR), and *p*-value for the comparison of the three groups was calculated by Kruskal–Wallis test. Categorical variables are described as frequencies and percentages, *p*-value was calculated by chi square test, and *p* ˂ 0.05 was considered to indicate statistical significance. Post hoc analysis with Bonferroni correction was performed for multiple hypothesis testing.

### 2.8. Propensity Score Matching

After selecting the patients and performing the analysis, we also performed propensity score matching by age and sex. We chose patients matched for sex and age in CTG and CNTG and performed the analysis again, similar to the original analysis. Therefore, we tried to explain that demographic differences were not the main cause of the different results between the two groups. This result is shown in Supplementary Tables S2 and S3.

## 3. Results

### 3.1. Basic Characteristics

The initial data contained those of 61,091 ED visits. We excluded visits related to nontreatment (e.g., for prescriptions, medical certificates) and visits cancelled without being seen (6561). In the final analysis, 31,793 patients were included in Phase 1 and 22,737 patients were included in Phase 2 in the final study, with 3223 CTG patients and 19,514 CNTG patients. Table 1 shows the distribution of the ED patients’ demographics. CTG patients were more likely to be older and predominantly male than CNTG or Phase 1 patients (mean age: CTG: 57.7 ± 20.7 years, Phase 1: 46.1 ± 25.5 years, CNTG: 48.3 ± 23.6 years) (Figure 1).

### 3.2. Outcome

CTG included more Korea Triage and Acuity Scale (KTAS) 1 and 2 patients than CNTG and Phase 2 (KTAS 1 and 2 patients (%); CNTG: 5.8%, CTG: 10.8%, CNTG: 5.8%). Compared to other groups, the rate of ambulance use was nearly 10% higher in CTG (Phase 1: 18.0%, CTG: 26.6%, CNTG: 17.3%, *p* < 0.001). The rate of admission was highest in CTG patients (CTG: 49.1%, CNTG: 75.4%, Phase 1: 73.5%). In addition, the ICU admission rate was higher in CTG than in the other two groups (CTG: 13.6%, Phase 1: 9.3%, Phase 2: 7.3%).

### 3.3. Process Changes in Overall Patients

**Figure 2:**
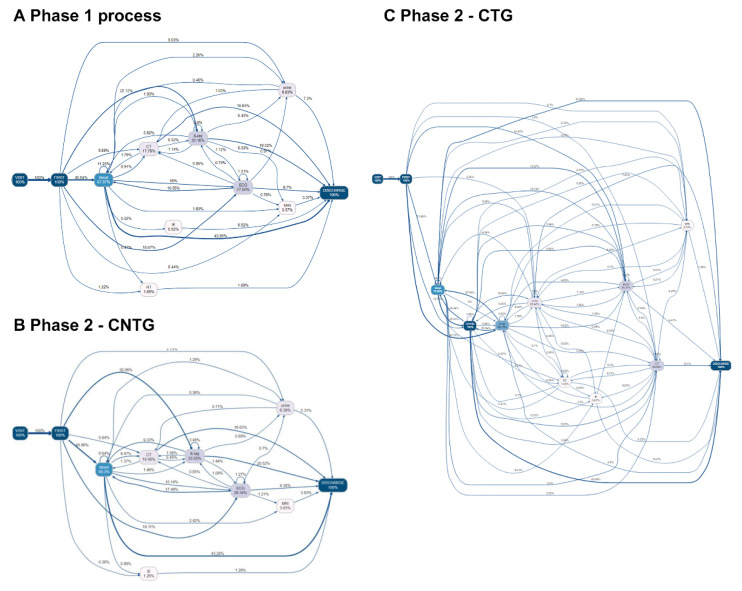
Process changes in ED patients before and after the COVID-19 pandemic. The top 65% of the most frequent process flow is shown in the figure. The arrow indicates process progression. The number above the line indicates the percentage of patients who followed the previous process to the process where the arrow points (%). Visit: time of the first visit to the ED. (**A**) Phase 1, (**B**) Phase 2-CTG, (**C**) Phase 2-CNTG. First: triage; IE: interventions and endoscopy; COVID-19: coronavirus disease; PCR: polymerase chain reaction; RT: nasal and sputum sample-based respiratory test; ECG: electrocardiography; Blood: blood sample-based laboratory tests; CT: computed tomography; discharge: time when the discharge order or the admission order was given.

**Figure 3:**
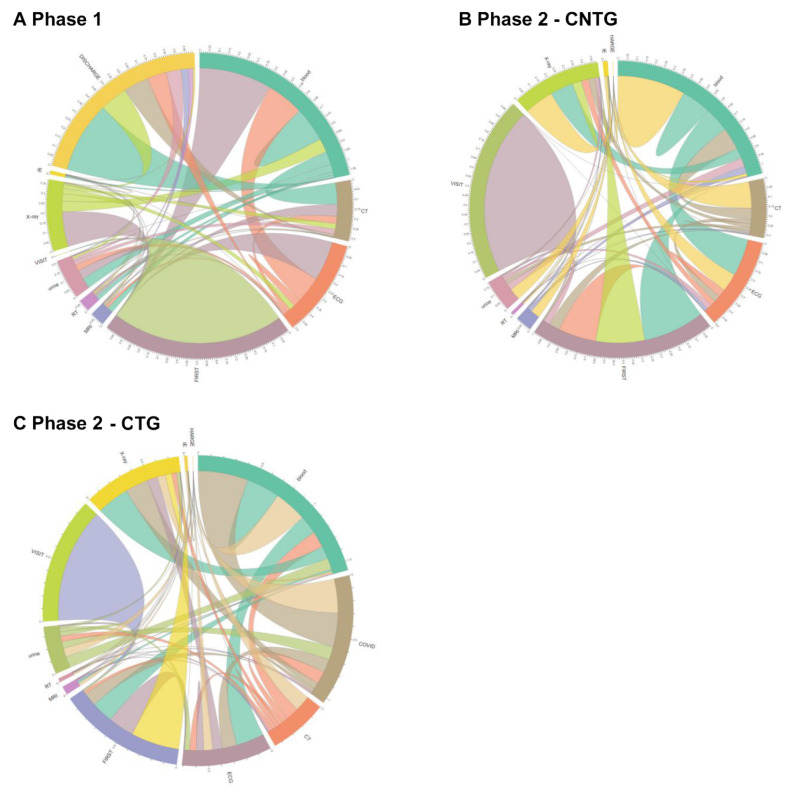
Chord diagram of process changes in the overall ED patients. The color refers to the next process, to which the patient moved. The number refers to the rate of patients who underwent the process. Visit: time of the first visit to the ED. (**A**) Phase 1, (**B**) Phase 2-CNTG, (**C**) Phase 2-CTG. First: triage; IE: interventions and endoscopy; COVID-19: coronavirus disease; PCR: polymerase chain reaction; RT: nasal and sputum sample-based respiratory rest; ECG: electrocardiography; Blood: blood sample-based laboratory tests; CT: computed tomography; Discharge: time when the discharge order or the admission order was given.

The number of activities performed per patient in Phase 1, CTG, and CNTG was 2.67, 5.93, and 2.57, respectively. Table 2 shows the difference in time consumption between each activity in three different groups: Phase 1, CTG, and CNTG. CTG patients took much more time on all occasions, and all the time gaps were significant at α = 0.05. Visit-to-triage time was higher in Phase 2 than in Phase 1 (Phase 1: 0.05 [0.02; 0.10] hours, CTG: 0.07 [0.03; 0.13] hours, CNTG: 0.05 [0.02; 0.12] hours). Overall time from visit to discharge was higher in CTG than in Phase 1 (Phase 1: 7.77 [4.47; 17.12], CTG: 14.76 [8.92; 22.24], CNTG: 6.98 [4.13; 13.28]). The process trace that starts from triage and proceeds directly to X-ray increased in CTG (48.56%) and CNTG (30.06%) more than in Phase 1 (22.12%) (Figure 2 and Figure 3). CTG patients underwent “COVID-19 PCR test.” However, the rate of triage to blood sample-based laboratory tests was lower in CTG than in Phase 1 and CNTG. Trace variety was increased in CTG (Figure 2). Post hoc analysis showed all processes were delayed in CTG, and a process of triage to X-ray was delayed in both CTG and CNTG.

### 3.4. Process Changes in Acute Cerebrovascular Patients

The number of activities performed per patient among patients with acute CVD in Phase 1, CTG, and CNTG was 5.54, 9.88, and 6.69, respectively, which is almost double that of the overall patients. In patients with acute CVD, median time from triage to CT (Phase 1: 1.04 h, CTG: 2.44 h, CNTG: 1.01 h) and median time from triage to MRI (Phase 1: 4.01 h, CTG: 5.54 h, CNTG: 3.47 h) were higher in CTG than in Phase 1. The median time from visit to triage time (Phase 1, 0.05 h; CTG, 0.05 h; CNTG, 0.05 h) and the total median time from visit to discharge (Phase 1, 10.28 h; CTG, 14.15 h; CNTG, 8.82 h) were also increased in CTG patients (Table 3).

**Figure 4:**
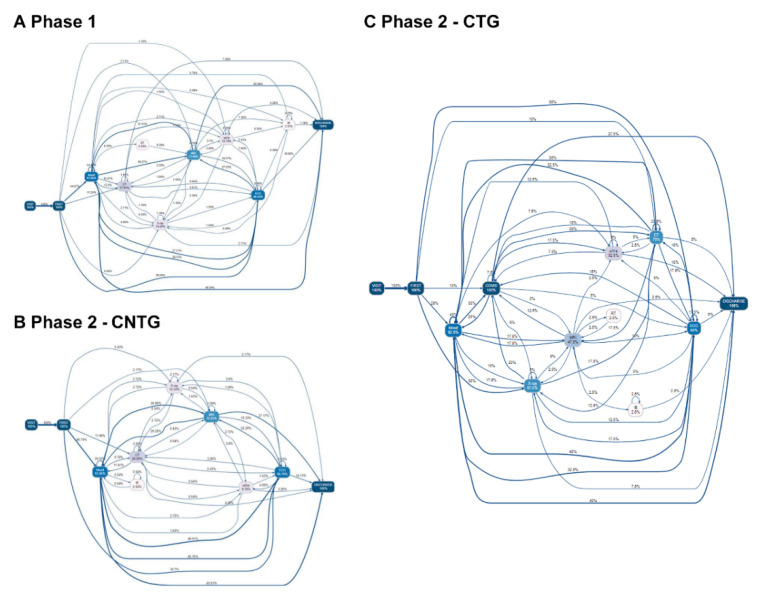
Process changes in patients with acute cerebrovascular disease before and after the COVID-19 pandemic. The top 65% of the process flow is shown in the figure. The arrow indicates process progression. The number above the line indicates the percentage of patients who followed the previous process to the process where the arrow points (%).Visit: time of the first visit to the ED; First: triage; IE: interventions and endoscopy; COVID-19: coronavirus disease; PCR: polymerase chain reaction; RT: nasal and sputum sample-based respiratory test; ECG: electrocardiography; Blood: blood sample-based laboratory tests; CT: computed tomography; discharge: time when the discharge order or the admission order was given. (**A**) Phase 1, (**B**) Phase 2-CTG, (**C**) Phase 2-CNTG.

**Figure 5:**
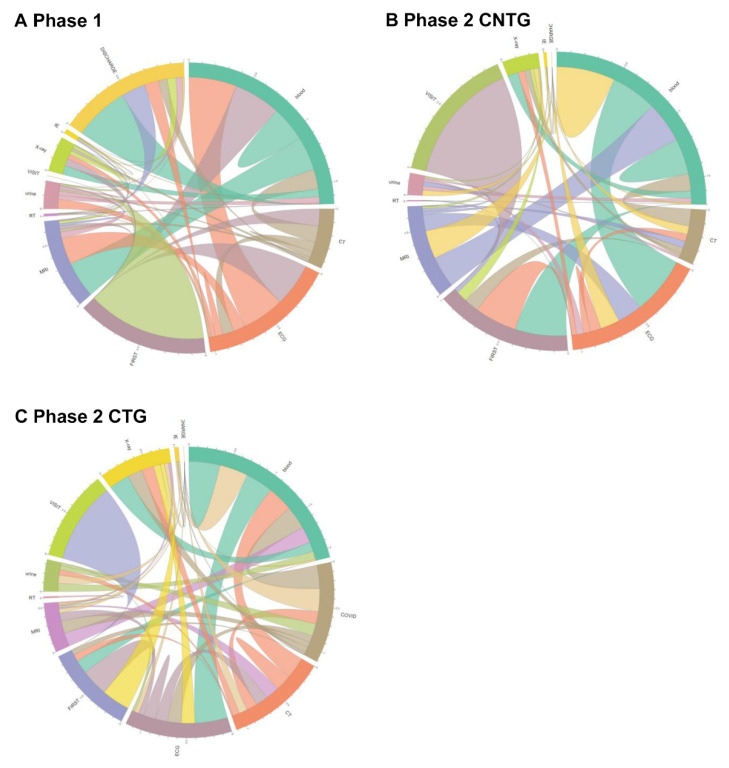
Chord diagram of overall ED patient process changes. The color indicates the next process that the patient moved to. The number refers to the rate of patients who underwent the process. (**A**) Phase 1, (**B**) Phase 2-CNTG, (**C**) Phase 2-CTG. Visit: time of the first visit to the ED; First: triage; IE: interventions and endoscopy; COVID-19: coronavirus disease; PCR: polymerase chain reaction; RT: nasal and sputum sample-based respiratory test; ECG: electrocardiography; Blood: blood sample-based laboratory tests; CT: computed tomography; Discharge: time when the discharge order or admission order was given.

In Phase 1, patients in the upper 65% trace normally undergo CT earlier than X-ray. However, in Phase 2, some of the CTG and CNTG patients underwent X-ray earlier than CT. Some patients even underwent X-ray immediately after the first triage step in CTG (30%) and CNTG (5.43%) (Figure 4 and Figure 5). Post hoc analysis showed the process from visit to triage was delayed in both CTG and CNTG, and those of CT and MRI from triage were delayed in CTG more than other groups.

### 3.5. Propensity Score Matching

We performed propensity score matching according to age and sex to balance the population of Phase 1, CTG, and CNTG to minimize selection bias. The results of propensity score matching showed the same pattern as that of the original population. Data of demographic characteristic and time difference are provided in Supplementary Tables S2–S4

## 4. Discussion

This study investigated not only the LOS in the ED but also the change in process using PM. According to the National Emergency Department Information System (NEDIS), mortality among patients who visited the ED increased after the COVID-19 outbreak in Korea [24]. The LOS of patients who had fever >37.5 °C also increased [24]. Our study also showed prolonged LOS in both overall ED and CVD patients. In addition, our study also compared delayed loop or process change before and after the pandemic to detect which process, namely, first visit to triage and triage to CT, was delayed in CTG group during a pandemic situation.

As shown in Figure 2 and Figure 4, the complexity of the ED process increased in COVID-19-tested patients. The COVID-19 test is a new process, and many process methods have been newly developed. The COVID-19 test itself was one of the process changes. As shown in Figure 2 and Figure 4, the incidence of direct first examination to X-ray process increased after the pandemic. In contrast, the first evaluation of blood decreased in COVID-19 patients. Before the pandemic situation, a blood test was performed first because intravenous (IV) line preparation is performed rapidly for imaging and other tests. This may be due to efforts to first rule out high-risk patients, such as performing chest radiography first to screen for pneumonia or performing COVID-19 PCR test before other evaluations, and minimize contacted medical staff from high-alert patients. Even in acute cerebrovascular patients, X-ray from the first evaluation increased.

In addition, the time from visit to triage completion increased in CTG and in both CTG and CNTG in propensity score matching. This means that time to triage increased compared to that before the pandemic, especially in CTG. To evaluate whether the patient was isolated from another patients for the COVID-19 test, the Center completed some questionnaires before admission and separated the patient into two triage stations: patients who had or who did not have a high risk of isolation for COVID-19. This questionnaire and separation might have caused the delay. However, it can also mean that a patient’s separation and triage itself is very important during a pandemic, and it can be one of the processes to focus on to improve the LOS and total process in the ED, for example, redistribution of highly trained people or experts to triage station or assigning more people for the administration of questionnaires.

Particularly, in acute CVD patients, we found that processes from visit to brain imaging, which is critical for patient diagnosis and outcome, were delayed. Process from visit to triage was delayed in both CTG and CNTG. Process from triage to brain imaging was delayed in CTG. In acute CVD, it is recommended to perform brain imaging as immediately as possible [14,25,26]. In case of another pandemic, this situation can be repeated, affecting a patient’s critical decision making, such as the use of tissue plasminogen activator therapy or intra-arterial therapy. This decision may be affected by hospital policies to isolate patients with high risk of infection diseases. On the other hand, the lack of resources made it impossible to perform high risk patients’ diagnosis with portable CT or MRI in isolated places. If possible, new devices should be prepared; however, it will be very expensive. The use of one existing imaging device (e.g., CT or MRI) separately, only for high-risk patients, and establishing a fast track for patients with these devices can be another option. In acute critical diseases, it will be important to establish another fast track during a pandemic to improve process and environment.

Each process time was delayed after COVID-19 compared to before, as determined from the COVID-19 PCR performed. This can be related to the studied center policy that a COVID-19-negative test result should be confirmed before MRI or other procedures are performed outside the ED in CTG group. However, in CTG patients, ECG, enzyme, and methods that can be performed without the COVID-19 test were also delayed. In addition, imaging (CT, MRI, ECG) to discharge, which is a process performed after the COVID-19 test and is not directly affected by COVID-19 PCR test time, was also delayed. Considering this, the COVID-19 test itself was not only a cause of delay in LOS after COVID-19 but also the pandemic situation itself.

In addition, despite the decrement of total ED visit patients per day, LOS was increased in CTG after COVID-19. Normally, it is well known that LOS increases when the number of ED patients increase, and this may mean that the effect of the COVID-19 event overwhelms the decrement of ED patients in the LOS of patients [8,27,28]. As explained above, our study supports that these results account for many process changes, such as the COVID-19 PCR test, X-ray, and prolonged triage.

All patients who visit the ED need and want proper time management. However, as shown in our study, in the COVID-19 pandemic situation, process and LOS in the ED change. All processes were delayed in CTG, and some processes, for example, process start from triage to x-ray or process start from first visit to triage in CVD patients, were delayed in CNTG. The best method will be prediction and prevention of the next pandemic; however, it is not always possible because, historically, the pandemic era has recurred [1,2]. Therefore, investigating whether delay is present and which process is delayed will be helpful to prepare for the next pandemic. Particularly, in acute critical disease, another fast track that is adjusted to a pandemic situation is needed [13]. Further study is needed to establish the exact strategy and protocol for the next pandemic.

### Limitation

This was a single-center retrospective study and there might be selection bias. To minimize bias, we compared the same period of patients before and after the COVID-19 pandemic. In addition, confounders were not considered between Phase 1 and Phase 2, for example, differences in the severity of patients or other patient demographic characteristics. However, we selected the same month of patients before and after COVID-19 and also divided Phase 2 again into those who underwent the COVID-19 test versus those who did not. Finally, there was a significant age difference between the two groups after selecting CTG and CNTG patients. We performed a propensity scoring test to show that there was no significant difference in the results despite adjusting for age between the two groups (Supplementary Tables S2 and S3).

In conclusion, after a pandemic outbreak, some processes were changed, some processes were newly developed, and there was no exception in acute cerebrovascular patients who needed proper time management. Therefore, if the next pandemic occurs, then the hospital should prepare a new process for a pandemic situation, based on previous experience, to minimize delay in ED patients.

## Figures and Tables

**Table 1:** Basic characteristics of the study population.

	Phase 1 (*n* = 31,793)	Phase 2 (*n* = 22,737)		
		CTG (*n* = 3223)	CNTG (*n* = 19,514)	*p*-Value
Age, median [IQR]	52 [26;66]	62 [46;73]	53 [31;66]	<0.001
Sex, *n* (%)				<0.001
Male	15,948 (50.2%)	1716 (53.2%)	9731 (49.9%)	
Female	15,845 (49.8%)	1507 (46.8%)	9783 (50.1%)	
SBP (mmHg), median [IQR]	121 [26;66]	125 [105;143]	128 [109;146]	<0.001
DBP (mmHg), median [IQR]	73 [61;84]	75 [62;87]	79 [66;90]	<0.001
PR (beats/min), median [IQR]	88 [76;104]	100 [85;116]	88 [76;102]	<0.001
RR (breaths/min), median [IQR]	18 [17;20]	18 [16;20]	18 [16;19]	<0.001
TEMP (°C), median [IQR]	36.9 [36.5;37.3]	37.3 [36.7;37.9]	36.8 [36.4;37.2]	<0.001
SpO_2_ (%), median [IQR]	98 [97;99]	97 [96;99]	98 [97;99]	<0.001
KTAS, *n* (%)				<0.001
1	189 (0.6%)	32 (1.0%)	70 (0.4%)	
2	1662 (5.2%)	316 (9.8%)	1049 (5.4%)	
3	14,486 (45.6%)	1723 (53.5%)	7775 (39.8%)	
4	14,207 (44.7%)	1041 (32.3%)	9349 (47.9%)	
5	1249 (3.9%)	111 (3.4%)	1270 (6.5%)	
Mental status, *n* (%)				<0.001
Alert	31,142 (98.0%)	3056 (94.8%)	19,183 (98.3%)	
Verbal	259 (0.8%)	61 (1.9%)	139 (0.7%)	
Pain	241 (0.8%)	76 (2.4%)	121 (0.6%)	
Unresponsive	151 (0.5%)	30 (0.9%)	70 (0.4%)	
Mode of arrival, *n* (%)				<0.001
Ambulance	5719 (18.0%)	858 (26.6%)	3376 (17.3%)	
Other	26,074 (82.0%)	2365 (73.4%)	16,137 (82.7%)	
Discharge, *n*(%)				<0.001
Home	23,377 (73.5%)	1381 (42.8%)	14,722 (75.4%)	
ED death	149 (0.5%)	31 (1.0%)	76 (0.4%)	
Transfer	1249 (3.9%)	228 (7.1%)	456 (2.3%)	
Admission	7018 (22.1%)	1583 (49.1%)	4260 (21.8%)	
Admission, *n* (%)				<0.001
ICU	657 (9.3%)	216 (13.6%)	312 (7.3%)	
GW	6361 (90.7%)	1367 (86.4%)	3948 (92.7%)	
Acute cerebrovascular disease	404 (1.2%)	60 (1.8%)	283 (1.4%)	0.012

Note: The *p*-values were calculated between all three groups (Phase1, CTG, and CNTG) by Kruskal–Wallis test for continuous variables and the chi^2^-test for categorical variables. Post hoc analysis was performed by Mann–Whitney test with Bonferroni adjustment. Result of post hoc analysis is shown in Supplementary Table S1. SBP: systolic blood pressure; DBP: diastolic blood pressure; PR: pulse rate; RR: respiratory rate; TEMP: temperature; SpO_2_: peripheral capillary oxygen saturation; KTAS: Korean Triage Acute Scale; CTG: COVID-19 tested Group; CNTG: COVID-19 not tested group; GW: general ward; ED: emergency department; ICU: intensive care unit.

**Table 2:** Number of activities and duration of each process in the overall patient population.

	^a^ Phase 1 (*n* = 84,017)	Phase 2 (*n* = 69,346)			
		^b^ CTG (*n* = 19,116)	^c^ CNTG (*n* = 50,230)	*p*-Value	Post-hoc Analysis
Overall, hours (median, [IQR])					
^α^ Visitto Triage	0.05 [0.02;0.10]	0.07 [0.03;0.13]	0.05 [0.02;0.12]	<0.001	c < a < b
Triage to CT	2.78 [1.40;4.16]	6.60 [3.24;10.72]	2.55 [1.27;4.10]	<0.001	c < a < b
Triage to MRI	4.70 [3.06;6.96]	12.03 [5.53;15.41]	4.37 [2.84;6.56]	<0.001	c < a < b
Triage to ECG	1.32 [0.49;2.95]	2.52 [0.91;5.19]	1.26 [0.59;2.68]	<0.001	a = c < b
Triage to X-ray	0.96 [0.51;2.53]	2.09 [0.91;5.19]	1.04 [0.56;2.41]	<0.001	a < c < b
Triage to ^β^ blood	1.77 [1.09;5.85]	5.15 [2.07;11.68]	1.71 [1.07;5.16]	<0.001	c < a < b
CT to discharge	4.39 [2.11;10.53]	7.59 [3.75;13.74]	3.67 [1.84;7.65]	<0.001	c < a < b
MRI to discharge	4.37 [2.26;11.11]	7.48 [3.84;14.02]	3.85 [2.01;7.42]	<0.001	c < a < b
ECG to discharge	4.90 [2.51;10.10]	9.54 [4.80;16.07]	4.36 [2.33;8.10]	<0.001	c < a < b
Visit to discharge	7.77 [4.47;17.12]	14.76 [8.92;22.24]	6.98 [4.13;13.28]	<0.001	c < a < b

Note: The *p*-values were calculated between the three groups (Phase1, CTG, and CNTG) by Kruskal–Wallis test for continuous variables. Post hoc analysis with Bonferroni correction was performed for multiple hypothesis testing. *n* refers to number of activities, which includes all tests and treatment performed on patients. ^α^ Visit: time of first visit to the ED. ^β^ Blood: blood sample-based laboratory tests. CNTG: COVID-19 Not Tested Group; CTG: COVID-19 Tested Group; CT: computed tomography; MRI: magnetic resonance imaging; SD: standard deviation; ECG: electrocardiography. In post hoc analysis, a refers to duration of process at phase 1, b refers to duration of process at CTG, and c refers to duration of CNTG.

**Table 3:** Number of activities and duration of each process in patients with presumed acute cerebrovascular disease.

	^a^ Phase 1 (*n* = 1978)	Phase 2 (*n* = 1840)			
		^b^ CTG (*n* = 488)	^c^ CNTG (*n* = 1352)	*p*-Value	Post-hoc Analysis
Acute cerebrovascular patients, hours (median, IQR)					
Visit to triage	0.05 [0.02;0.10]	0.05 [0.03;0.12]	0.05 [0.03;0.13]	<0.001	a < b = c
Triage to CT	1.04 [0.46;2.91]	2.44 [1.07;6.71]	1.01 [0.55;2.08]	<0.001	a = c < b
Triage to MRI	4.01 [2.63;5.74]	5.54 [3.73;12.00]	3.47 [2.30;5.34]	<0.001	a = c < b
Triage to blood	2.32 [1.08;7.03]	4.04 [1.29;14.41]	1.87 [0.90;5.68]	<0.001	c < a < b
CT to discharge	5.69 [3.10;10.54]	9.85 [4.03;16.80]	4.47 [2.88;8.39]	0.001	a = c < b
MRI to discharge	5.89 [3.01;11.02]	9.44 [5.10;12.15]	4.81 [2.80;7.98]	0.001	c < a = b
Visit to discharge	10.28 [6.35;18.68]	14.15 [9.48;22.83]	8.82 [5.34;12.45]	<0.001	c < a < b

Note: The *p-values* were calculated between the three groups (Phase1, CTG, and CNTG) using the Kruskal–Wallis test for continuous variables. Post hoc analysis with Bonferroni correction was performed for multiple hypothesis testing. The *n* refers to number of activities, which includes all tests and treatment performed on patients. CNTG: COVID-19 Not Tested Group; CTG: COVID-19 Tested Group; CT: computed tomography; MRI: magnetic resonance imaging; SD: standard deviation. In post hoc analysis, a refers to duration of process at phase 1, b refers to duration of process at CTG, c refers to duration of CNTG.

## Supplementary Materials

The following are available online at https://www.mdpi.com/2077-0383/9/12/3842/s1. Figure S1: Questionnaire used to distinguish candidates of COVID-19 test in triage. In question 6, a daily updated list of places where large-scale COVID-19 outbreaks arose. Table S1: Post hoc analysis of basic characteristics of the study population between three groups. Table S2: Basic characteristics after propensity score matching. Table S3: Duration of each process after propensity score matching. Table S4: Post hoc analysis of basic characteristics of the study population between three groups after propensity score matching.
