# Supplementary material for: Impact of COVID-19 Pandemic on the Overall Diagnostic and Therapeutic Process for Patients of Emergency Department and Those with Acute Cerebrovascular Disease
Source: J Clin Med. 2020 Nov 26;9(12):3842. doi: 10.3390/jcm9123842 (PMC7760535; doi:10.3390/jcm9123842)
Supplement: Supplementary file 1 [file jcm-09-03842-s001.pdf]

Journal of
Clinical Medicine

MDPI

Supplementary Materials

Screening Questionnaire for COVID-19

※ Please complete with nothing but the truth to questions about COVID-19.
- False statement or deliberate concealment of facts can be a major hindrance to the patient, as well as to medical institutions and society as a whole. In accordance with Article 35, Clause 2 of the Infectious Disease-Related Laws, a false statement is subject to a fine up to 10 million KRW.

Patient Name		Patient #	

1. Check all current symptoms.

☐ Cold symptoms (cough, phlegm, sore throat, rhinorrhea, myalgia, lost sense of smell or taste, dyspnea)
☐ Fever above 37.5 ˚C
☐ Cold symptoms (cough, phlegm, sore throat, rhinorrhea, myalgia, lost sense of smell or taste, dyspnea) + Fever above 37.5 ˚C
☐ No Symptoms

2. Have you or your family members been required to self-quarantine for being in contact with a person with COVID-19? (Self-quarantine due to overseas travel history is not applicable.)

☐ Yes ➔ If "yes" go to question ① & ②
① Who has been contacted as a self-quarantine?
☐ Yourself ☐ Live-in family member ☐ Yourself and live-in family member
② When were you and/or live-in family member released from self-quarantine?
(Released date from self- quarantine:_______/_______)
☐ No ➔ If "No" go to question 5

3. Have you ever been diagnosed with COVID-19?

☐ Yes (Released date of quarantine:_______/_______)
☐ No

4. Have you ever done COVID-19 test?

☐ Yes, I did and it was negative
☐ Yes, I did and I am waiting for the results
☐ Never done

5. Have you or live-in family member recently travelled overseas?

☐ Yes ➔ If "yes" go to question ① & ② & ③
① Who travelled?
☐ Patient ☐ Live-in family member ☐ Patient and live-in family member
② Name of country: ____________________
③ When did you and/or live-in family member arrive in Korea? (Arrival date:_____/_____/_____)
☐ No ➔ If "No" go to question 6

1

(→ Please keep writing on the back.)

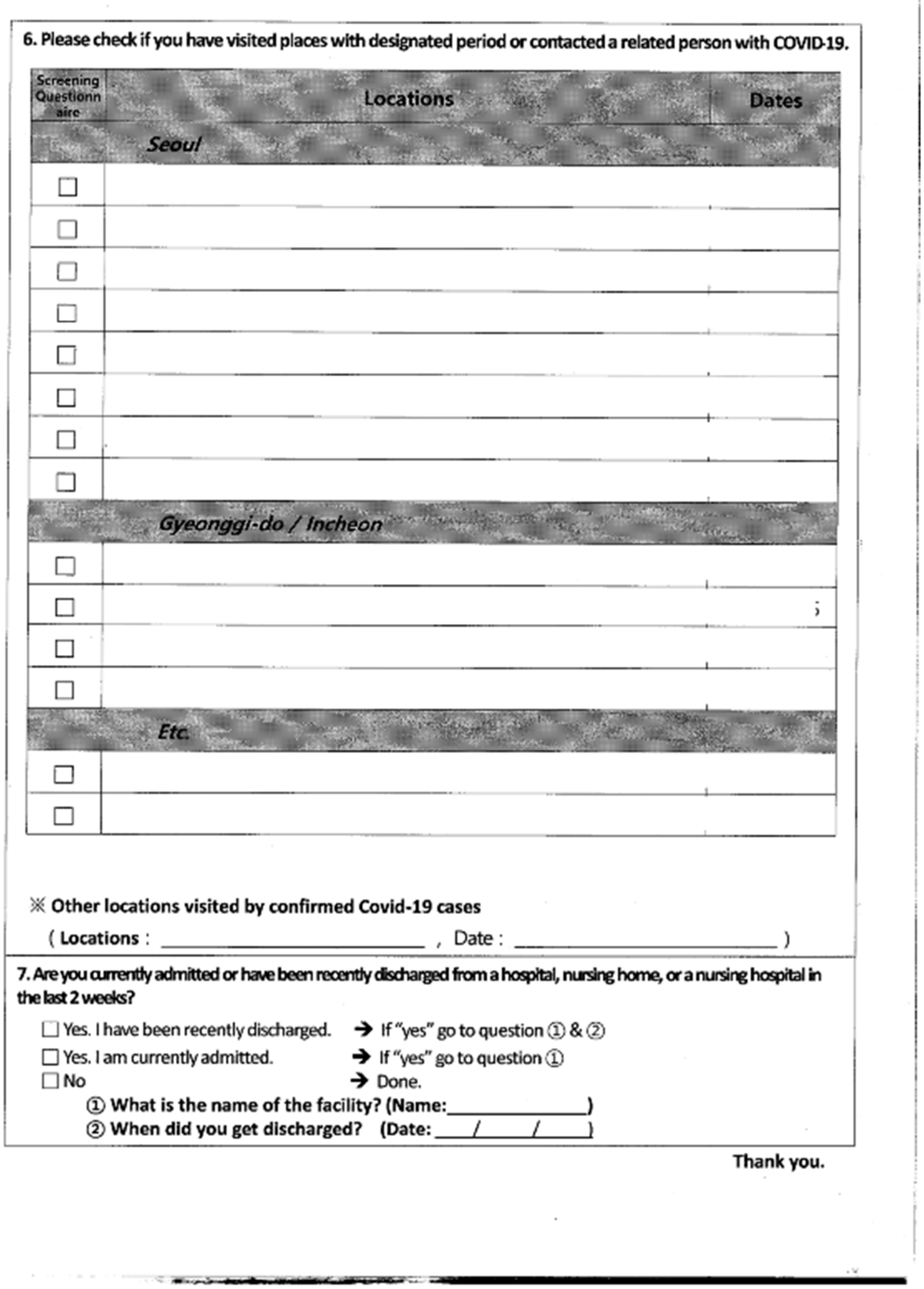

6. Please check if you have visited places with designated period or contacted a related person with COVID-19.

Screening Questionnaire	Locations	Dates
	Seoul	
☐		
☐		
☐		
☐		
☐		
☐		
☐		
☐		
	Gyeonggi-do / Incheon	
☐		
☐		
☐		
☐		
	Etc.	
☐		
☐		

※ **Other locations visited by confirmed Covid-19 cases**
(**Locations** : ________________________ , Date : ________________________)

7. Are you currently admitted or have been recently discharged from a hospital, nursing home, or a nursing hospital in the last 2 weeks?

☐ Yes. I have been recently discharged. → If "yes" go to question ① & ②
☐ Yes. I am currently admitted. → If "yes" go to question ①
☐ No → Done.
① **What is the name of the facility? (Name:______________)**
② **When did you get discharged? (Date:____/____/____)**

Thank you.

Supplementary Figure S1. Questionnaire used for distinguish candidate of COVID-19 test in triage: In questionnaire 6, a daily updated list of places where large-scale COVID-19 outbreaks arose.

Supplementary Table S1. Post-hoc analysis of basic characteristics of the study population between three groups.

Variable		Groups	*p*-value	Post-hoc Analysis
Age		Phase1 vs CTG vs CNTG	<0.001	a < c < b
Sex	Male	Phase1 vs CTG	0.001	a < b
		Phase1 vs CNTG	0.001	a < c
		CTG vs CNTG	0.5220	
	Female	Phase1 vs CTG	0.001	a < b = c
		Phase1 vs CNTG	0.001	

		CTG vs CNTG	0.5220	
SBP (mmHg), median [IQR]		Phase1 vs CTG vs CNTG	<0.001	a < b < c
DBP (mmHg), median [IQR]		Phase1 vs CTG vs CNTG		a < b < c
PR (beats/min), median [IQR]		Phase1 vs CTG vs CNTG		a = c < b
RR (breaths/min), median [IQR]		Phase1 vs CTG vs CNTG		c < a = b
TEMP (°C), median [IQR]		Phase1 vs CTG vs CNTG		c < a < b
SpO_2 (%), median [IQR]		Phase1 vs CTG vs CNTG		b < a < c
KTAS	1	Phase1 vs CTG	0.009	c < a < b
		Phase1 vs CNTG	<0.001	
		CTG vs CNTG	<0.001	
	2	Phase1 vs CTG	<0.001	a = c < b
		Phase1 vs CNTG	0.479	
		CTG vs CNTG	<0.001	
	3	Phase1 vs CTG	<0.001	c < a < b
		Phase1 vs CNTG	<0.001	
		CTG vs CNTG	<0.001	
	4	Phase1 vs CTG	<0.001	b < a < c
		Phase1 vs CNTG	<0.001	
		CTG vs CNTG	<0.001	
	5	Phase1 vs CTG	0.191	a = b < c
		Phase1 vs CNTG	<0.001	
		CTG vs CNTG	<0.001	
Mental status	Alert	Phase1 vs CTG	<0.001	b < a < c
		Phase1 vs CNTG	0.0053	
		CTG vs CNTG	<0.001	
	Verbal	Phase1 vs CTG	<0.001	c = a < b
		Phase1 vs CNTG	0.218	
		CTG vs CNTG	<0.001	
	Pain	Phase1 vs CTG	<0.001	c = a < b
		Phase1 vs CNTG	0.078	
		CTG vs CNTG	<0.001	
	Unresponse	Phase1 vs CTG	0.001	c < a < b
		Phase1 vs CNTG	0.059	
		CTG vs CNTG	<0.001	
Mode of Arriaval	Ambulance	Phase1 vs CTG	<0.001	a = c < b
		Phase1 vs CNTG	0.05	
		CTG vs CNTG	<0.001	
Discharge	Home	Phase1 vs CTG	<0.001	b < a < c
		Phase1 vs CNTG	<.0001	
		CTG vs CNTG	<0.001	
	ED Death	Phase1 vs CTG	<0.001	a = c < b
		Phase1 vs CNTG	0.212	
		CTG vs CNTG	<0.001	
	Transfer	Phase1 vs CTG	<0.001	c < a < b
		Phase1 vs CNTG	<0.001	
		CTG vs CNTG	<0.001	
	Admission	Phase1 vs CTG	<0.001	a = c < b
		Phase1 vs CNTG	0.525	
		CTG vs CNTG	<0.001	
ICU		Phase1 vs CTG	<0.001	c < a < b
		Phase1 vs CNTG	<0.001	
		CTG vs CNTG	<0.001	
GW		Phase1 vs CTG	<0.001	c = a < b
		Phase1 vs CNTG	<0.001	
		CTG vs CNTG	0.540	
Acute Cerebrovascular disease patient		Phase1 vs CTG	0.019	a < b
		Phase1 vs CNTG	0.093	
		CTG vs CNTG	0.093	

SBP: systolic blood pressure; DBP: diastolic blood pressure; PR: pulse rate; RR: respiratory rate; TEMP: temperature; SpO_2: peripheral capillary oxygen saturation; KTAS: Korean Triage Acute Scale; CTG: COVID-19 PCT Tested Group; CNTG: COVID-19 PCR not tested group; GW: general ward; ED: emergency department; ICU: intensive care unit. a refers to duration of process at phase 1, b refers to duration of process at CTG, c refers to duration of CNTG

Supplementary Table S2. Basic Characteristics after Propensity score matching.

	Phase1[a] (*n* = 2985)	Phase2 (*n* = 5985)		
		CTG [b] (*n* = 3223)	CNTG [c] (*n* = 2762)	*p*-value
Age, median [IQR]	62 [48;73]	62 [46;73]	63 [48;73]	0.251
Sex, *n* (%)				0.961
Male	1600 (53.6%)	1716 (53.2%)	1475 (53.4%)	
Female	1385 (46.4%)	1507 (46.8%)	1287 (46.6%)	
SBP (mmHg), median [IQR]	127 [110;144]	125 [105;143]	133 [115;151]	<0.001
DBP (mmHg), median [IQR]	75 [65;85]	75 [62;87]	80 [69;92]	<0.001
PR (beats/min), median [IQR]	87 [75;101]	100 [85;116]	87 [76;101]	<0.001
RR (breaths/min), median [IQR]	18 [16;20]	18 [16;20]	18 [16;19]	<0.001
TEMP (°C), median [IQR]	36.8 [36.5;37.2]	37.3 [36.7;37.9]	36.7 [36.4;37.1]	<0.001
SPO2 (%), median [IQR]	98 [97;99]	97 [96;99]	98 [97;99]	<0.001
KTAS, *n* (%)				<0.001
1	24 (0.8%)	32 (1.0%)	15 (0.5%)	
2	191 (6.4%)	316 (9.8%)	138 (5.0%)	
3	1408 (47.2%)	1723 (53.5%)	1146 (41.5%)	
4	1267 (42.4%)	1041 (32.3%)	1295 (46.9%)	
5	95 (3.2%)	111 (3.4%)	168 (6.1%)	
AVPU, *n* (%)				<0.001
Alert	2892 (96.9%)	3056 (94.8%)	2700 (97.8%)	
Verbal	41 (1.4%)	61 (1.9%)	22 (0.8%)	
Pain	29 (1.0%)	76 (2.4%)	24 (0.9%)	
Unresponsive	23 (0.8%)	30 (0.9%)	16 (0.6%)	
ER Method, *n*(%)				<0.001
Ambulance	633 (21.2%)	858 (26.6%)	528 (19.1%)	
Other	2262 (78.8%)	2365 (73.4%)	2234 (80.9%)	
ER Route, *n*(%)				<0.001
Direct	2319 (77.7%)	2287 (71.0%)	2226 (80.6%)	
Other	576 (22.3%)	936 (29.0%)	536 (19.4%)	
ER Result, *n*(%)				<0.001
Home	2039 (68.3%)	1381 (42.8%)	2003 (72.5%)	
ER Death	19 (0.6%)	31 (1.0%)	14 (0.5%)	
Transfer	156 (5.2%)	228 (7.1%)	71 (2.6%)	
Admission	771 (25.8%)	1583 (49.1%)	674 (24.4%)	
Admission, *n*(%)				<0.001
ICU	100 (12.9%)	216 (13.6%)	69 (10.2%)	
General ward	671 (87.1%)	1367 (86.4%)	605 (89.8%)	

The *p*-values are calculated between all three groups (Phase1, CTG and CNTG) by Kruskal-Wallis test for continuous variables and the chi-square test for categorical variables. Post-hoc analysis was performed by Mann-Whitney test with Bonferroni adjustment. Result of post-hoc analysis is shown in Supplementary Table S1. AVPU: alert, verbal, pain, unresponsive. a refers to duration of process at phase 1, b refers to duration of process at CTG, c refers to duration of CNTG.

Supplementary Table S3. Duration of each process after Propensity score matching.

	Phase1 [a] (*n = 9172)	Phase2 (*n* = 27,040)			
		CTG [b]	CNTG [c]	*p-value*	Post-hoc

		(*n* = 19,116)	(*n* = 7924)		Analysis
Overall, time (median [IQR])					
$^{\alpha}$ Visit to Triage	0.05 [0.02;0.10]	0.07 [0.03;0.13]	0.05 [0.02;0.12]	<0.001	a < c < b
Triage to CT	2.94 [1.67;4.37]	6.60 [3.24;10.72]	2.65 [1.39;4.17]	<0.001	a = c < b
Triage to MRI	5.01 [3.07;7.83]	12.03 [5.53;15.41]	4.30 [2.65;6.66]	<0.001	c < a < b
Triage to ECG	1.33 [0.45;3.17]	2.52 [0.91;5.19]	1.40 [0.62;2.98]	<0.001	a = c < b
Triage to X-ray	1.22 [0.60;3.67]	2.09 [0.91;5.19]	1.16 [0.61;2.69]	<0.001	a = c < b
Triage to $^{\beta}$ blood	1.94 [1.09;7.66]	5.15 [2.07;11.68]	1.87 [1.10;6.11]	<0.001	a = c < b
CT to Discharge	4.74 [2.33;11.48]	7.59 [3.75;13.74]	3.77 [1.96;7.89]	<0.001	c < a < b
MRI to Discharge	4.48 [2.37;11.44]	7.48 [3.84;14.02]	4.11 [2.28;7.56]	<0.001	a = c < b
ECG to Discharge	5.52 [2.65;10.74]	9.54 [4.80;16.07]	4.44 [2.37;7.93]	<0.001	c < a < b
Visit to Discharge	9.13 [5.20;19.33]	14.76 [8.92;22.24]	7.37 [4.53;14.97]	<0.001	c < a < b

P-values are calculated between the three groups (Phase1, CTG and CNTG) by Kruskal-Wallis test for continuous variables. Post-hoc analysis with Bonferroni correction were performed for multiple hypothesis testing. * n refers to number of activities which includes all tests and treatment performed to patients. $^{\alpha}$ Visit: time of first visit to the ED. $^{\beta}$ Blood: blood sample-based laboratory tests. In post-hoc analysis, a refers to duration of process at phase 1, b refers to duration of process at CTG, c refers to duration of CNTG

Supplementary Table S4. Post-hoc analysis of basic characteristics of the study population between three groups after propensity scoring matching.

Variable		Groups	*p*-value	Post-hoc Analysis
Age, median [IQR]				a = b = c
Sex	Male	Phase1 vs. CTG	0.922	a = b = c
		Phase1 vs. CNTG	0.922	
		CTG vs. CNTG	0.922	
	Female	Phase1 vs. CTG	0.922	a = b = c
		Phase1 vs. CNTG	0.922	
		CTG vs. CNTG	0.922	
SBP (mmHg), median [IQR]				b < a < c
DBP (mmHg), median [IQR]				a = b < c
PR (beats/min), median [IQR]				a = c < b
RR (breaths/min), median [IQR]				c < a < b
TEMP (°C), median [IQR]				c < a < b
SPO2 (%), median [IQR]				b < a = c
KTAS	1	Phase1 vs. CTG	0.207	a = b = c
		Phase1 vs. CNTG	0.514	
		CTG vs. CNTG	0.446	
	2	Phase1 vs. CTG	<0.001	a = c < b
		Phase1 vs. CNTG	0.025	
		CTG vs. CNTG	<0.001	
	3	Phase1 vs. CTG	<0.001	c < a < b
		Phase1 vs. CNTG	<0.001	
		CTG vs. CNTG	<0.001	
	4	Phase1 vs. CTG	<0.001	b < a < c
		Phase1 vs. CNTG	<0.001	
		CTG vs. CNTG	<0.001	
	5	Phase1 vs. CTG	0.615	a = b < c
		Phase1 vs. CNTG	<0.001	
		CTG vs. CNTG	<0.001	
AVPU	Alert	Phase1 vs. CTG	<0.001	b < a < c
		Phase1 vs. CNTG	0.005	
		CTG vs. CNTG	<0.001	
	Verbal	Phase1 vs. CTG	0.132	c < b
		Phase1 vs. CNTG	0.072	
		CTG vs. CNTG	0.001	
	Pain	Phase1 vs. CTG	<0.001	a = c < b

		Phase1 vs. CNTG	0.788	
		CTG vs. CNTG	<0.001	
	Unresponse	Phase1 vs. CTG	0.584	a = b = c
		Phase1 vs. CNTG	0.584	
		CTG vs. CNTG	0.480	
Mode of Arriaval	Ambulance	Phase1 vs. CTG	<0.001	c < a < b
		Phase1 vs. CNTG	0.0357	
		CTG vs. CNTG	<0.001	
Discharge	Home	Phase1 vs. CTG	<0.001	b < a < c
		Phase1 vs. CNTG	<0.001	
		CTG vs. CNTG	<0.001	
	ED Death	Phase1 vs. CTG	0.296	a = b = c
		Phase1 vs. CNTG	0.635	
		CTG vs. CNTG	0.180	
	Transfer	Phase1 vs. CTG	<0.001	c < a < b
		Phase1 vs. CNTG	<0.001	
		CTG vs. CNTG	<0.001	
	Admission	Phase1 vs. CTG	<0.001	a = c < b
		Phase1 vs. CNTG	0.224	
		CTG vs. CNTG	<0.001	
ICU		Phase1 vs. CTG	<0.001	a = c < b
		Phase1 vs. CNTG	0.067	
		CTG vs. CNTG	<0.001	
GW		Phase1 vs. CTG	<0.001	a = c < b
		Phase1 vs. CNTG	0.623	
		CTG vs. CNTG	<0.001	
